# Tick Surveillance for Relapsing Fever Spirochete *Borrelia miyamotoi* in Hokkaido, Japan

**DOI:** 10.1371/journal.pone.0104532

**Published:** 2014-08-11

**Authors:** Ai Takano, Kochi Toyomane, Satoru Konnai, Kazuhiko Ohashi, Minoru Nakao, Takuya Ito, Masako Andoh, Ken Maeda, Masahisa Watarai, Kozue Sato, Hiroki Kawabata

**Affiliations:** 1 Department of Veterinary Medicine, Joint Faculty of Veterinary Medicine, Yamaguchi University, Yamaguchi, Yamaguchi, Japan; 2 The United Graduate School of Veterinary Science, Yamaguchi University, Yamaguchi, Yamaguchi, Japan; 3 Department of Disease Control, Graduate School of Veterinary Medicine, Hokkaido University, Sapporo, Hokkaido, Japan; 4 Department of Parasitology, Asahikawa Medical College, Asahikawa, Hokkaido, Japan; 5 Department of Infectious Disease, Hokkaido Institute of Public Health. Sapporo, Hokkaido, Japan; 6 Laboratory of Veterinary Public Health, Joint Faculty of Veterinary Medicine, Kagoshima University, Kagoshima, Kagoshima, Japan; 7 Department of Bacteriology-I, National Institute of Infectious Diseases, Shinjuku-ku, Tokyo, Japan; 8 United Graduate School of Agricultural Science & Veterinary Science, Gifu University, Gifu, Gifu, Japan; University of Kentucky College of Medicine, United States of America

## Abstract

During 2012–2013, a total of 4325 host-seeking adult ticks belonging to the genus *Ixodes* were collected from various localities of Hokkaido, the northernmost island of Japan. Tick lysates were subjected to real-time PCR assay to detect borrelial infection. The assay was designed for specific detection of the Relapsing fever spirochete *Borrelia miyamotoi* and for unspecific detection of Lyme disease-related spirochetes. Overall prevalence of *B. miyamotoi* was 2% (71/3532) in *Ixodes persulcatus*, 4.3% (5/117) in *Ixodes pavlovskyi* and 0.1% (1/676) in *Ixodes ovatus*. The prevalence in *I. persulcatus* and *I. pavlovskyi* ticks were significantly higher than in *I. ovatus*. Co-infections with Lyme disease-related spirochetes were found in all of the tick species. During this investigation, we obtained 6 isolates of *B. miyamotoi* from *I. persulcatus* and *I. pavlovskyi* by culture in BSK-M medium. Phylogenetic trees of *B. miyamotoi* inferred from each of 3 housekeeping genes (*glpQ*, *16S rDNA*, and *flaB*) demonstrated that the Hokkaido isolates were clustered with Russian *B. miyamotoi*, but were distinguishable from North American and European *B. miyamotoi*. A multilocus sequence analysis using 8 genes (*clpA*, *clpX*, *nifS*, *pepX*, *pyrG*, *recG*, *rplB*, and *uvrA*) suggested that all Japanese *B. miyamotoi* isolates, including past isolates, were genetically clonal, although these were isolated from different tick and vertebrate sources. From these results, *B. miyamotoi*-infected ticks are widely distributed throughout Hokkaido. Female *I. persulcatus* are responsible for most human tick-bites, thereby *I. persulcatus* is likely the most important vector of indigenous relapsing fever from tick bites in Hokkaido.

## Introduction

*Borrelia miyamotoi*, a member of the relapsing fever group (RF) borreliae, was first discovered from *Ixodes persulcatus* ticks and the rodent, *Apodemus argenteus*, in Hokkaido, the northernmost island of Japan [1]. Subsequently, *B. miyamotoi* has been found in *Ixodes scapularis* and *Ixodes pacificus* ticks in North America [2], [3], [4] and *Ixodes ricinus* in Europe [5], [6]. Cases of human infections with *B. miyamotoi* were initially reported in Russia [7]. Following this report, several human cases have been confirmed as *B. miyamotoi* infections in North America [8], [9] and Europe [10]. Recently, tick surveillance for *B. miyamotoi* was performed in Europe [11] and Russia [7]. The surveys showed that *I. ricinus* and *I. persulcatus* in the Eurasian continent consistently harbor *B. miyamotoi* with low prevalence. However, large-scale tick surveillance has not been conducted in Asian countries where *I. persulcatus* is distributed.

In Japan, cases of Lyme disease (LD) are clustered in the northernmost island, Hokkaido, because its principal vector, *I. persulcatus*, is abundant throughout the island [12], [13]. The tick is also a candidate vector for *B. miyamotoi* [1]. A previous study in Hokkaido detected *B. miyamotoi* from *I. persulcatus* ticks and from rodents [14]. However, widespread prevalence data of *B. miyamotoi* infections are still lacking for populations of ticks, including *I. persulcatus* and other genus *Ixodes* species. This basic information on prevalence of *B. miyamotoi* infections in ticks is urgently required for risk assessment of relapsing fever in Hokkaido.

*Ixodes pavlovskyi*, a species closely related to *I. persulcatus*, is also distributed throughout Hokkaido [15], [16], [17]. Compared to the abundant *I. persulcatus*, *I. pavlovskyi* is a minor tick species. Nevertheless, the close evolutionary relationship among *I. persulcatus* and *I. pavlovskyi* [16], [17] raises concerns about their ability to maintain *B. miyamotoi* in nature. In this study, large-scale tick surveillance for *B. miyamotoi* was conducted in Hokkaido to estimate the infection rate of host-seeking adult *Ixodes* ticks. The tick-derived isolates of *B. miyamotoi* established in this surveillance were subjected to molecular analyses to characterize their genetic profile. The resultant field and laboratory data will serve as a baseline in understanding the epidemiology of *B. miyamotoi* in Japan.

## Materials and Methods

### Tick collection, DNA extraction and borrelial cultivation

**Figure 1:**
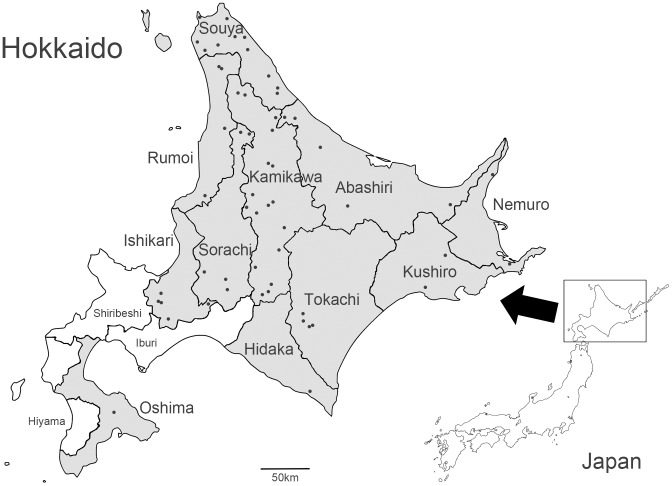
Tick collection sites in this study. Ticks were collected in the areas shown by black dots. The gray shading shows district where ticks were collected during this investigation (see Table 1).

During the spring to summer seasons (April to July) of 2012 and 2013, host-seeking adult ticks of *I. persulcatus*, *I. pavlovskyi* and *I. ovatus* were collected by flagging over vegetation from a total of 57 forested areas in Hokkaido (Figure 1). In these areas, no specific permissions were required for collection of ticks, and this study did not involve endangered or protected species. The collection of *I. ovatus* was discontinued in 2013 because of its extremely low prevalence of *B. miyamotoi* infection. The number of ticks examined, and the prevalence of *Borrelia* spp. among them were calculated for each district.

All of the ticks collected were subjected to a quantitative real-time PCR (qPCR) assay to detect spirochete DNA. The DNA for PCR templates was prepared from whole or half bodies of each tick by using sodium hydroxide (NaOH), as described previously [18]. Briefly, tick tissues including bacterial cells were lysed in 50 µL of 25 mM NaOH for 10 minutes at 95°C. After adding 4 µL of Tris-HCL (1 M, pH7.5) for neutralization, the lysate was centrifuged at 4°C, and the resulting supernatant was used as template DNA for qPCR. A preliminary study suggested that the sensitivity of qPCR using the lysate was equal to that using DNA extracted by DNeasy Tissue Kit (Qiagen, CA, USA) (Data not shown).

Parts of ticks collected were used to cultivate *B. miyamotoi* in modified Barbour-Stoenner-Kelly medium (BSK-M: using minimal essential medium alpha [BioWest, Germany] as a substitute for CMRL-1066) under microaerophilic conditions [19], [20] (Table S1). The surface of ticks was sterilized by washing with 0.1% sodium hypochlorite, then with 80% ethyl alcohol. The washed tick was longitudinally bisected using a disposable knife (ELP No. 10, Akiyama Medical MFG. CO., LTD., Tokyo, Japan), and one half was inoculated into BSK-M medium. The remaining half was used to prepare the PCR template, as described above. Tick samples which were shown by qPCR to be positive for *B. miyamotoi* and negative for LD borreliae, were cultivated at 30°C for 4 weeks, and then the growth of spirochetes was examined by dark-field microscopy every two weeks.

### Detection of borrelial DNAs from ticks

As described previously [21], borrelial DNA in the tick lysates was detected by multiplex qPCR targeting the 16 S rRNA gene (*16 S rDNA*). Based on Barbour et al., a minor groove binder probe with an FAM-label (FAM probe) (Life Technologies Corporation, MD, USA) was designed for LD borreliae, while an minor groove binder probe with a VIC-label (VIC probe) was designed for RF borreliae, including *B. miyamotoi*. The qPCR was performed using Premix Ex *Taq* (Probe qPCR), (Takara Bio Inc., Shiga, Japan) according to the manufacturer's instructions. The qPCR was run on an ABI PRISM 7000 system, ABI StepOne system (Life Technologies Corporation), and LightCycler 480 systemII (Roche Diagnostics, Basel, Switzerland). The PCR cycles were set at 40, and the reaction was performed in 12.5 µL (ABI PRISM 7000 system and StepOne system) or 10 µL (LightCycler 480 systemII) in single tubes or plate wells. The sensitivity and specificity of the qPCR was performed as described in the File S1.

### PCR, multilocus sequence analysis (MLSA), and phylogeny reconstruction

Tick-derived isolates of *B. miyamotoi* were characterized by PCR-based DNA sequencing. After DNA extraction, using Wizard genomic DNA purification kit (Promega, WI, USA), Takara Ex *Taq* (Takara Bio Inc.) and KOD FX (TOYOBO Co., LTD., Osaka, Japan) DNA polymerases were used for all PCR amplifications, as recommended by the manufacturers. Target DNA fragments of the borrelial flagellin gene (*flaB*), glycerophosphoryl diester phosphodiesterase gene (*glpQ*), and *16 S rDNA* were amplified as described previously [22]. MLSA was performed to further characterize the isolates of *B. miyamotoi*. For this purpose, the loci from 8 genes (*clpA*, *clpX*, *nifS*, *pepX*, *pyrG*, *recG*, *rplB*, and *uvrA*) were analyzed [23]. These primers were designed by genome assembling of 4 RF borreliae; *Borrelia hermsii* (CP000048), *Borrelia duttonii* (CP000976), *Borrelia turicatae* (CP000049), and *Borrelia recurrentis* (CP000993). The PCR thermal conditions to amplify the 8 genes were as follows: 95°C for 30 s, followed by 35 cycles of 95°C for 10 s, 50°C for 30 s, and 72°C for 60 s. All PCR products were purified by High Pure PCR Product Purification Kit (Roche Diagnostics), then directly sequenced using an ABI Prism 3130 or 3130xl Genetic Analyzer (Life Technologies Corporation). All of the PCR primers used in this study are listed in the Table S2.

Raw sequence data were assembled by Sequencher 5.1 (Gene Codes Corporation, MI, USA). Sequences of each gene were aligned by ClustalW (v.1.6), and Neighbor-joining trees were generated by 1000 bootstrap repetitions under the Kimura 2-parameter by MEGA 5.2 [22], [24]. All positions containing alignment gaps and missing nucleotides were eliminated only in pairwise sequence comparisons (the pairwise deletion option was used).

### Statistical tests for prevalence data

Prevalence data of borrelial infections in tick groups from different categories (e.g. species, sex and locale) were analyzed by the Fisher's exact tests. A p-value less than 0.01 was considered to be statistically significant.

## Results

### Sensitivity and specificity of the qPCR

The sensitivity and specificity of the qPCR were examined, as shown in the supporting information. The limit of the DNA detection consistently observed was a minimum of 10 plasmid copies (data not shown). Regarding the specificity of qPCR, in the case of the FAM probe, 85% (11/13) of LD borreliae were detected, whereas *Borrelia valaisiana* Am501^T^ was barely detectable, *Borrelia lusitaniae* PotiB2^T^ was not detected, and none of the RF borreliae were detected. On the other hand, the VIC probe detected *B. miyamotoi*, *B. hermsii*, and *Borrelia coriaceae* Co53^T^. However, for species with a 1 bp mismatch with the probe, such as *B. duttonii* Ly and *Borrelia* sp. AGRF [25], amplification was delayed, resulting in a reduced final concentration that just barely reached our threshold. None of the reptile associated borreliae were detected by either probe. Therefore, the FAM probe widely detects *B. burgdorferi* sensu lato, which has been isolated from *Ixodes* ticks in Japan, including pathogenic borreliae of humans, such as *B. garinii, Borrelia bavariensis* and *Borrelia afzelii*, and non-pathogenic borreliae, *Borrelia japonica*, *Borrelia tanuki* and *Borrelia turdi*, whereas the VIC probe detects a narrow range of relapsing fever borreliae, of which the only one reported in Japan is *B. miyamotoi*.

### Prevalence of borreliae in *Ixodes* ticks

**Figure 2:**
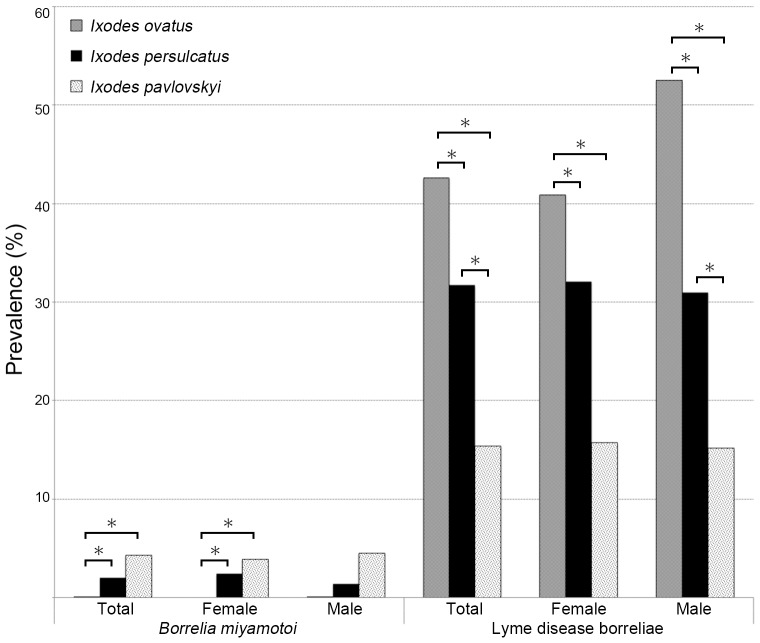
The prevalence of *B. miyamotoi* and LD borreliae in *Ixodes* ticks in Hokkaido. The asterisk shows significant difference: * P<0.01, by Fisher's exact test.

A total of 4325 adult ticks were collected from various localities covering 11 out of the total 14 districts of Hokkaido (Figure 1). As shown in Table 1, ticks harboring *B. miyamotoi* were found from 8 districts in northern, eastern and central Hokkaido. There was no significant statistical difference in the prevalence among the 8 localities. Overall prevalence of *B. miyamotoi* in ticks of Hokkaido was 2% in *I. persulcatus*, 4.3% in *I. pavlovskyi*, and 0.1% in *I. ovatus,* respectively (Table 1). There was no statistical difference between *I. persulcatus* and *I. pavlovskyi*. The prevalences among both of these tick species were statistically higher than that of *I. ovatus* (Figure 2). The overall prevalence of LD borreliae was 31.7% in *I. persulcatus*, 15.4% in *I. pavlovskyi*, and 42.6% in *I. ovatus*, respectively (Table 1). Co-infections of *B. miyamotoi* and LD borreliae in adult ticks were observed in 25 of 3532 *I. persulcatus* (0.7%), 1 of 117 *I. pavlovskyi* (0.9%), and 1 of 676 *I. ovatus* (0.1%).

**Table 1:** Prevalence of borreliae in ticks.

Location in Hokkaido		Tick species	Tick Number			*B. miyamotoi* positive No. (%)*			LD borreliae positive No. (%)*		
			Male	Female	Total	Male	Female	Total	Male	Female	Total
North	Souya	*I. persulcatus*	223	266	489	6 (2.7)	5 (1.9)	11 (2.2)	42 (18.8)	61 (22.9)	103 (21.1)
		*I. pavlovskyi*	1	0	1	0 (0)	0 (0)	0 (0)	0 (0)	0 (0)	0 (0)
	Rumoi	*I. persulcatus*	13	14	27	0 (0)	0 (0)	0 (0)	4 (30.8)	5 (35.7)	9 (33.3)
		*I. ovatus*	3	21	24	0 (0)	0 (0)	0 (0)	1 (33.3)	11 (52.4)	12 (50)
	Kamikawa	*I. persulcatus*	499	986	1485	5 (1)	20 (2)	25 (1.7)	165 (33.1)	308 (31.2)	473 (31.9)
		*I. pavlovskyi*	25	24	49	1 (4)	2 (8.3)	3 (6.1)	7 (28)	4 (16.7)	11 (22.4)
		*I. ovatus*	40	401	441	1 (2.5)	0 (0)	1 (0.2)	18 (45)	144 (35.9)	162 (36.7)
East	Abashiri	*I. persulcatus*	250	280	530	7 (2.8)	9 (3.2)	16 (3)	107 (42.8)	123 (43.9)	230 (43.4)
		*I. pavlovskyi*	0	3	3	0 (0)	0 (0)	0 (0)	0 (0)	1 (33.3)	1 (33.3)
		*I. ovatus*	12	54	66	0 (0)	0 (0)	0 (0)	7 (58.3)	32 (59.3)	39 (59.1)
	Nemuro	*I. persulcatus*	91	95	186	1 (1.1)	2 (2.1)	3 (1.6)	24 (26.4)	23 (24.2)	47 (25.3)
	Kushiro	*I. persulcatus*	143	186	329	0 (0)	6 (3.2)	6 (1.8)	60 (42)	79 (42.5)	139 (42.2)
	Tokachi	*I. persulcatus*	76	72	148	1 (1.3)	3 (4.2)	4 (2.7)	17 (22.4)	21 (29.2)	38 (25.7)
		*I. pavlovskyi*	0	3	3	0 (0)	0 (0)	0 (0)	0 (0)	0 (0)	0 (0)
		*I. ovatus*	44	101	145	0 (0)	0 (0)	0 (0)	26 (59.1)	49 (48.5)	75 (51.7)
Central	Sorachi	*I. persulcatus*	67	73	140	0 (0)	2 (2.7)	2 (1.4)	6 (9)	13 (17.8)	19 (13.6)
	Hidaka	*I. persulcatus*	7	9	16	0 (0)	0 (0)	0 (0)	1 (14.3)	1 (11.1)	2 (12.5)
	Ishikari	*I. persulcatus*	63	116	179	0 (0)	4 (3.4)	4 (2.2)	14 (22.2)	35 (30.2)	49 (27.4)
		*I. pavlovskyi*	40	21	61	2 (5)	0 (0)	2 (3.3)	3 (7.5)	3 (14.3)	6 (9.8)
South	Oshima	*I. persulcatus*	2	1	3	0 (0)	0 (0)	0 (0)	0(0)	0(0)	0(0)
Total		*I. persulcatus*	1434	2098	3532	20 (1.4)	51 (2.4)	71 (2)	444 (31)	674 (32.1)	1118 (31.7)
		*I. pavlovskyi*	66	51	117	3 (4.5)	2 (3.9)	5 (4.3)	10 (15.2)	8 (15.7)	18 (15.4)
		*I. ovatus*	99	577	676	1 (1)	0 (0)	1 (0.1)	52 (52.5)	236 (40.9)	288 (42.6)

*The numbers include mixed infections.

### Phylogenetic analysis of isolated strains

**Figure 3:**
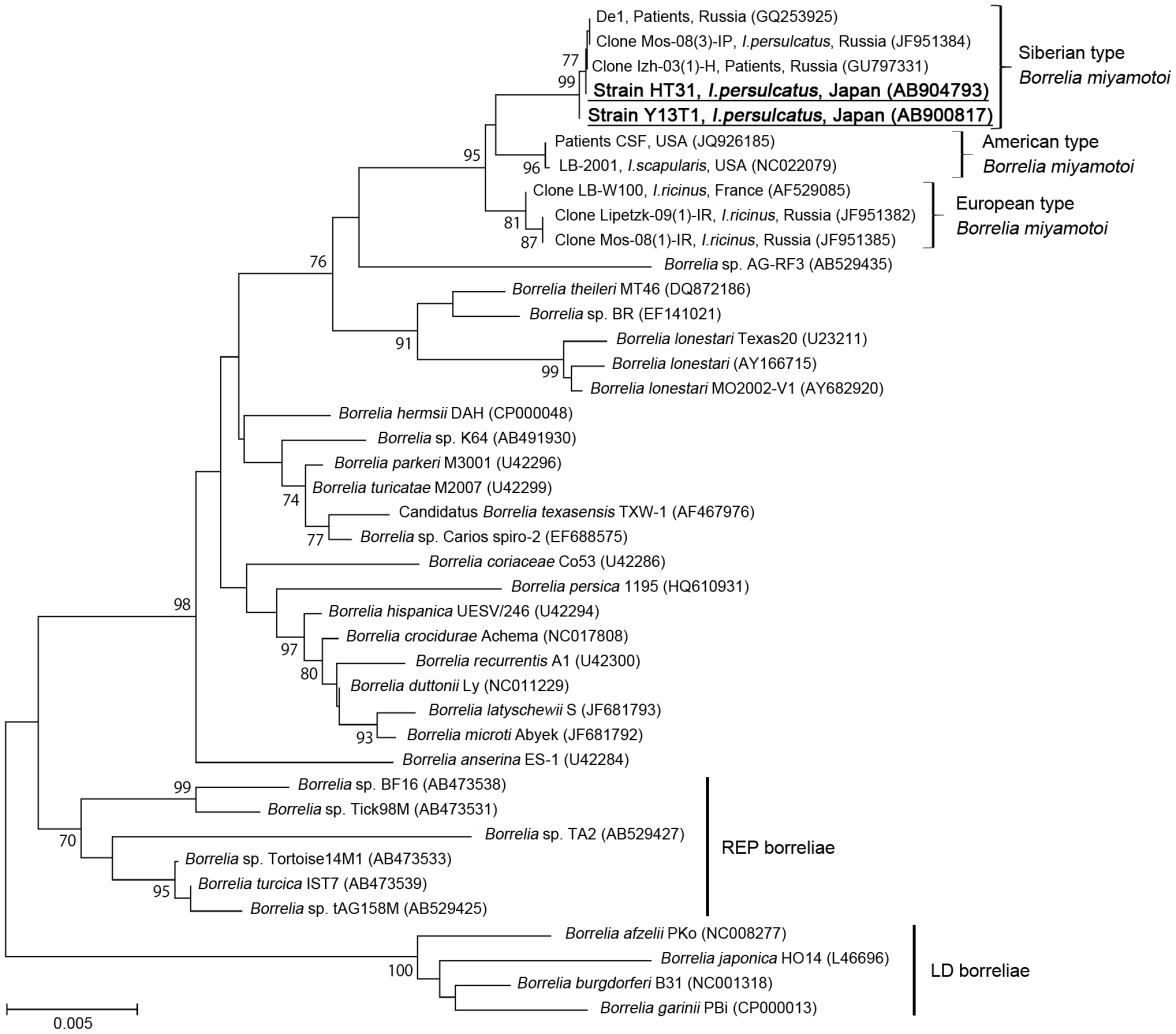
Phylogenetic analysis of RF borreliae based on *16 S rDNA* of *Borrelia* spp. The phylogenetic tree of *16 S rDNA* was constructed. The phylogenetic branches were supported in >70% by the bootstrap analysis. The bar indicates the percentage of sequence divergence. Sequences in this study were shown in bold type. If possible, clone or strain name, isolation source, and country were described in the case of *B. miyamotoi*. Numbers in parentheses indicate Accession Numbers in GenBank.

In this study, 6 isolates of *B. miyamotoi* derived from *I. persulcatus* and *I. pavlovskyi* were established in culture using BSK-M medium (Table 2). These isolates were utilized for molecular characterization, together with 5 isolates previously established in Hokkaido [1]. In the initial step of molecular analyses, DNA sequences of housekeeping genes (*glpQ*, *16 S rDNA,* and *flaB*) were determined for all of the Hokkaido isolates, and there were no nucleotide substitutions in all 11 isolates. Therefore, the isolate HT31 (the type strain of *B. miyamotoi*) was selected as a representative for phylogenetic analysis. Neighbor-joining phylogenetic trees inferred from each of the housekeeping genes clearly showed that HT31 was clustered with *B. miyamotoi* from Russia, but was distinguishable from *B. miyamotoi* found in North American and *B. miyamotoi* detected from most *I. ricinus* ticks in Europe (Figures 3, S1, and S2). These three clusters of *B. miyamotoi* were temporally designated as “Siberian,” “American” and “European” types, in this study (Figure 3).

**Table 2:** *Borrelia miyamotoi* strains used in this study.

Strain name	Isolation source	Location	Isolated year	Reference
HT31^T^	*I. persulcatus*, female	Shiretoko, Hokkaido, Japan	1990	1
HT24	*I. persulcatus*, female	Shiretoko, Hokkaido, Japan	1990	1
FR64b	*Apodemus argenteus*, whole blood	Furano, Hokkaido, Japan	1991	1
Hk004	*I. persulcatus*, nymph	Sibecha, Hokkaido, Japan	1993	1
NB103-1	*I. persulcatus*, nymph on *Emberiza spodocephala*	Nemuro, Hokkaido, Japan	1991	1
MYK1	*I. pavlovskyi*, female	Shibetsu, Hokkaido, Japan	2012	in this study
MYK2	*I. persulcatus*, female	Nayoro, Hokkaido, Japan	2012	in this study
MYK3	*I. persulcatus*, female	Obihiro, Hokkaido, Japan	2012	in this study
MYK4	*I. persulcatus*, female	Simukapp, Hokkaido, Japan	2012	in this study
MYK5	*I. persulcatus*, female	Takinoue, Hokkaido, Japan	2012	in this study
Y13T1	*I. persulcatus*, male	Oumu, Hokkaido, Japan	2013	in this study

The molecular typing by MLSA using 8 genes (*clpA*, *clpX*, *nifS*, *pepX*, *pyrG*, *recG*, *rplB,* and *uvrA*) revealed that all of the Hokkaido isolates were identical to each other. Sequence similarity between the Hokkaido isolates (Siberian type) and American type of *B. miyamotoi* (LB-2001 [26], Acc. No. NC_022079) were 98% in concatenated sequences of the 8 genes (4776 bp), but the values ranged from 97.5 to 99.1% in individual genes. A phylogenetic tree constructed using concatenated sequences is shown in the supporting information (Figure S3). Nucleotide sequences of *B. miyamotoi* isolates from Hokkaido were deposited in DDBJ/EMBL/GenBank databases as the following accession numbers: AB900797–807 and AB904793 for HT31^T^, AB900808–16 for MYK1, and AB900817 for Y13T1.

## Discussion

It is known that *I. persulcatus* principally serves as a tick vector for LD borreliae in Hokkaido. Hokkaido has an area of approximately 83,000 km^2^, and the total population of Hokkaido, according to the National Census 2010, is 5.5 million people (http://www.stat.go.jp/english/index.htm). However, 42% of the population inhabits the plains of Ishikari district (Figure 1). Similar to the taiga zone in Russia, *I. persulcatus* ticks are abundant mainly in forested areas of northern and eastern Hokkaido, which are thinly populated. Human tick bites and tick-borne LD, therefore, occur sporadically in Hokkaido [27]. In this study, we conducted large-scale tick surveillance for *B. miyamotoi* in Hokkaido, because human cases of *B. miyamotoi* infections have been confirmed in Russia [7]. Moreover, human *B. miyamotoi* infection, determined through retrospective analysis, has been reported in Hokkaido (http://www.nih.go.jp/niid/ja/relapsing-fever-m/relapsing-fever-iasrs/3877-pr4046.html). Our study demonstrated that *B. miyamotoi* is widely distributed throughout Hokkaido with low prevalence (0–3%) in *I. persulcatus* (Table 1, Figure 2). The prevalence is similar to those reported in Russian and European *I. persulcatus* [7], [11]. Besides *I. persulcatus*, *B. miyamotoi* was found from 4.3% (within 0–6.1%) of *I. pavlovskyi*, and this is the first report of detection and isolation of *B. miyamotoi* from *I. pavlovskyi*. Moreover, we detected *B. miyamotoi* from an *I. ovatus* male. However, given that the prevalence in *I. ovatus* was significantly lower than that in *I. persulcatus* and *I. pavlovskyi*, the potential vectors of *B. miyamotoi* in Hokkaido are thought to be *I. persulcatus* and *I. pavlovskyi*. Female ticks of *I. persulcatus* are responsible for most human tick-bites in Hokkaido [27]. The abundance of *B. miyamotoi*-infected female ticks is, therefore, directly linked to human risk. Although the prevalence of *B. miyamotoi* is relatively low in rural communities of Hokkaido, *B. miyamotoi* is important for public health as an agent of fever and variable symptoms. Moreover, *I. pavlovsky*i can serve as an additional vector for *B. miyamotoi*. *I. persulcatus* and *I. pavlovskyi* are closely related morphologically, ecologically and phylogenetically [17], [28]. The larvae and nymphs of both tick species have a wide range of feeding hosts, such as small mammals and birds. On the other hand, unlike *I. persulcatus*, adult *I. pavlovskyi* mainly infests birds. The potential for *I. pavlovskyi* to bite humans is still unclear, however, its public health significance is negligible given the far lower abundance.

MLSA is an efficient tool to resolve phylogenetic relationships of LD borreliae on an intraspecies level [23]. In this study, we obtained for MLSA, 11 isolates, including 5 strains isolated in the early 1990's. These isolates were 100% identical in 8 loci, even from different tick and vertebrate sources. This result suggests that Japanese *B. miyamotoi* are clonal. The reason, however, remains unclear. Further analysis, such as the examination of MLSA using European *B. miyamotoi* or *B. miyamotoi* from the main island of Japan may contribute to the elucidation of its evolutionary history, e.g. clonal expansion.

In previous studies, suspected human cases of co-infection with *B. miyamotoi* and LD borreliae have been reported from North America and Russia [6], [7]. In this study, we also found co-infection of *B. miyamotoi* and LD borreliae in 3 tick species. Of 1118 *I. persulcatus* ticks which were infected with LD borreliae, 25 (2.2%) were co-infected with *B. miyamotoi*. It, therefore, seems likely that approximately 2% of LD patients might be co-infected with *B. miyamotoi* in Hokkaido. On the other hand, it is unclear when, during feeding, *B. miyamotoi* is transmitted to vertebrates through saliva. Whereas LD borreliae are known to require several days (∼48 hours) for transmission from ticks to vertebrates [29], soft tick-borne RF borreliae were efficiently transmitted through saliva within minutes [30]. In a previous study, we demonstrated that *Borrelia* sp. AGRF, which is phylogenetically related to *B. miyamotoi*, is maintained in the salivary gland of unfed hard-ticks, suggesting that the borrelial transmission to vertebrate hosts occurs rapidly when the ticks start to feed blood [25]. Thus, we speculate that *B. miyamotoi* may be transmitted to humans more rapidly than LD borreliae, thereby increasing the risk of infection. Further studies will be required to confirm this.

In conclusion, the present surveillance showed that *B. miyamotoi*-infected ticks are widely distributed throughout Hokkaido, and that *I. persulcatus* is likely the most important vector of indigenous relapsing fever-like spirochetes transmitted by ticks in Hokkaido. Our subsequent genetic analyses also demonstrated that the Hokkaido isolates of *B. miyamotoi* are clonal.

## Supporting Information

Figure S1 **Phylogenetic analysis of RF borreliae based on** ***flaB*** **of** ***Borrelia*** **spp.** The phylogenetic branches were supported in >70% by the bootstrap analysis. The bar indicates the percentage of sequence divergence. Sequences in this study are shown in bold type. The number in parentheses indicates Accession Number in GenBank.
(TIF)

Figure S2 **Phylogenetic analysis of RF borreliae based on** ***glpQ*** **of** ***Borrelia*** **spp.** The phylogenetic branches were supported in >70% by the bootstrap analysis. The bar indicates the percentage of sequence divergence. Sequences in this study are shown in bold type. The number in parentheses indicates Accession Number in GenBank.
(TIF)

Figure S3 **Bayesian phylogenetic inference of concatenated housekeeping gene sequences of RF borreliae.** The phylogenetic tree was constructed based on Bayesian phlylogenetic inference as previously described by Margos et al [23]. The posterior probability values of the clades are provided. Bars labeled 0.02 depict 2% divergence. The LD borreliae (ST1 [*B. burgdorferi* B31], ST84 [*B. garinii* PBi], ST70 [*B. afzelii* VS461] were downloaded from the MLST website; www.mlst.net) were used as outgroups (data not indicated). The number in parentheses indicates Accession Number in GenBank.
(TIF)

Table S1 The composition of BSK-M medium.
(DOC)

Table S2 The Primer list used in this study.
(DOC)

File S1 The sensitivity and specificity of the qPCR.
(DOC)
