# Supplementary figures and images for: Tick Surveillance for Relapsing Fever Spirochete Borrelia miyamotoi in Hokkaido, Japan
Source: PLoS One. 2014 Aug 11;9(8):e104532. doi: 10.1371/journal.pone.0104532 (PMC4128717; doi:10.1371/journal.pone.0104532)

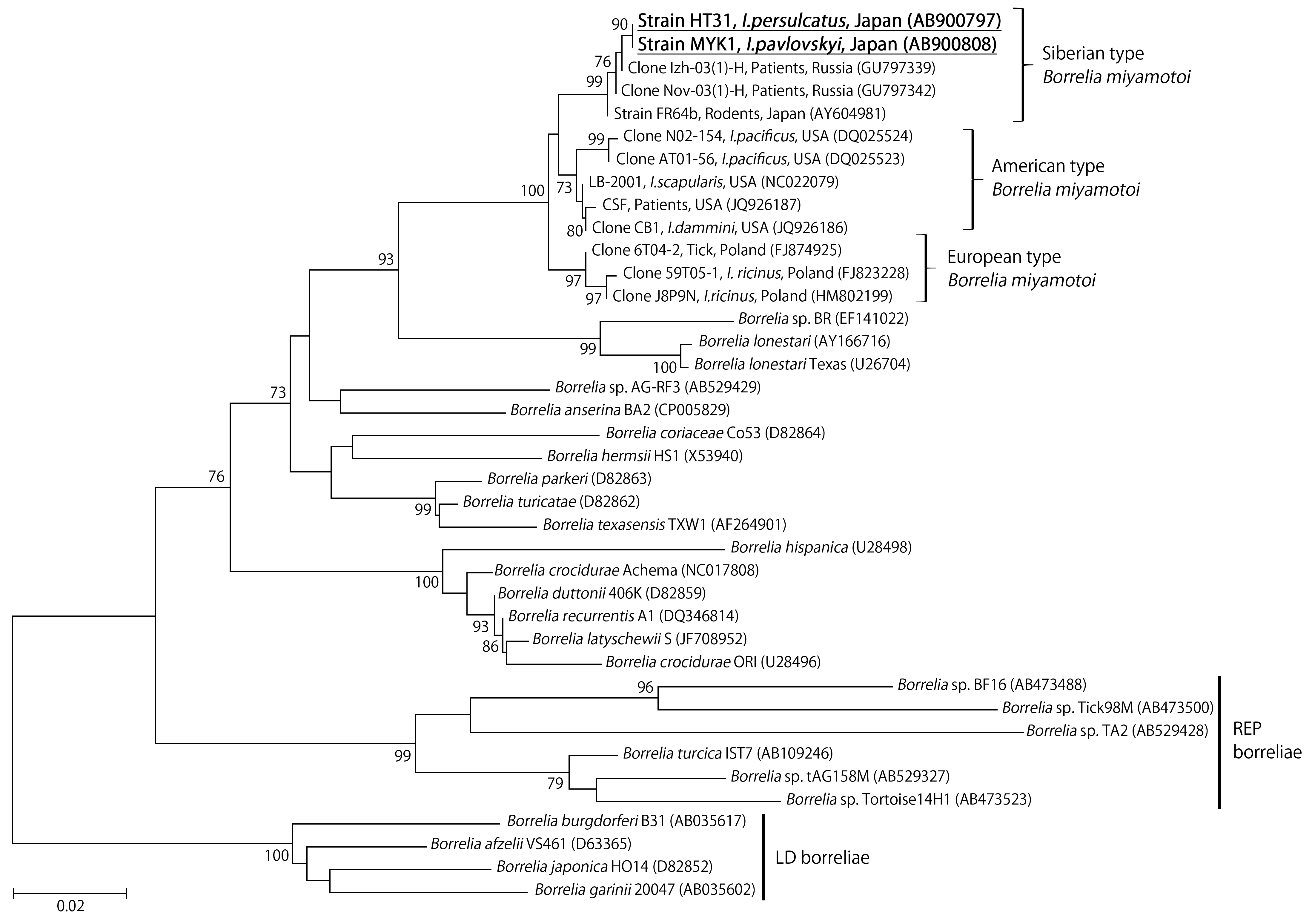

Supplement: Figure S1 — Phylogenetic analysis of RF borreliae based on flaB of Borrelia spp. The phylogenetic branches were supported in >70% by the bootstrap analysis. The bar indicates the percentage of sequence divergence. Sequences in this study are shown in bold type. The number in parentheses indicates Accession Number in GenBank. (TIF) [file pone.0104532.s001.tif]

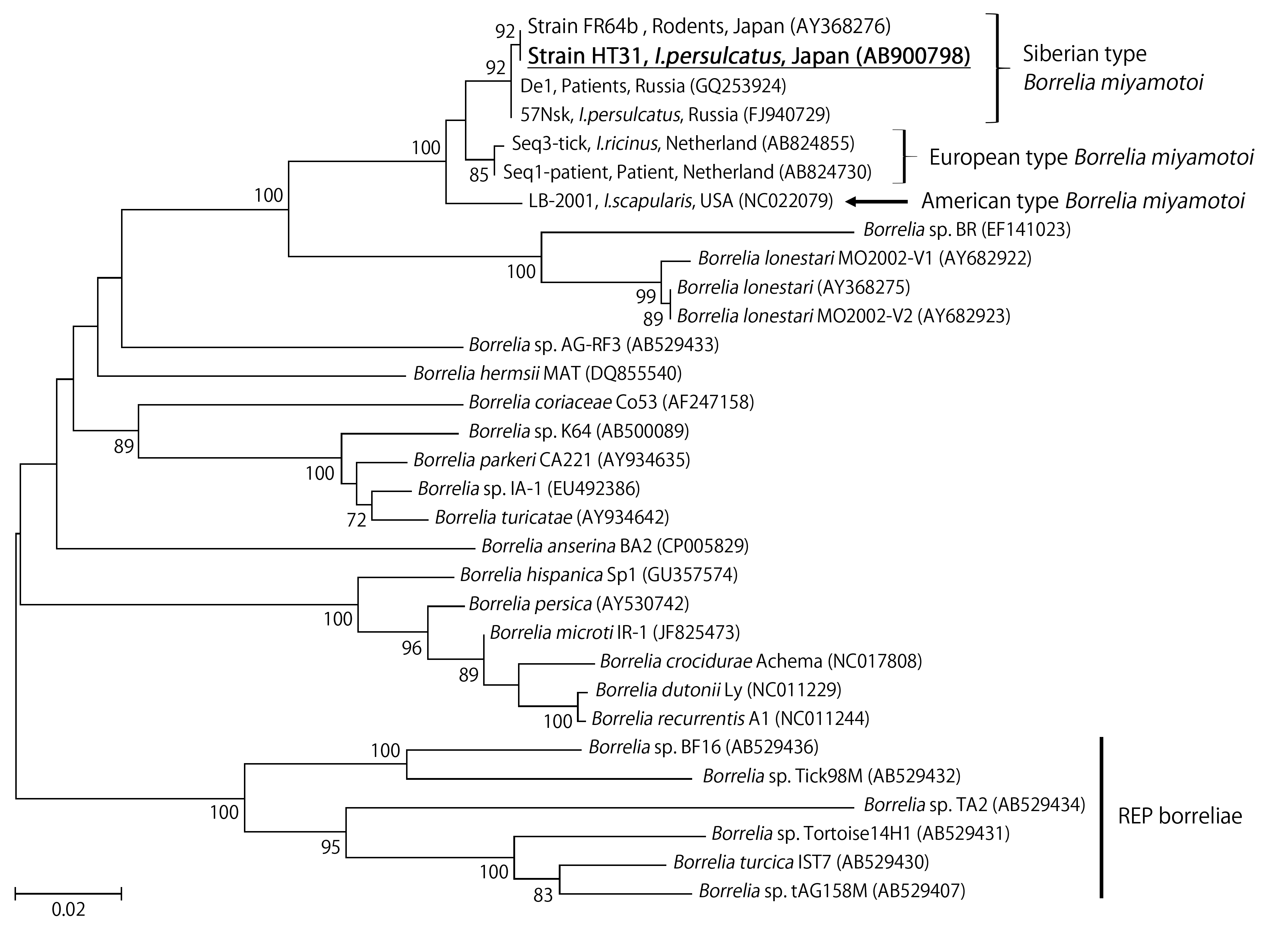

Supplement: Figure S2 — Phylogenetic analysis of RF borreliae based on glpQ of Borrelia spp. The phylogenetic branches were supported in >70% by the bootstrap analysis. The bar indicates the percentage of sequence divergence. Sequences in this study are shown in bold type. The number in parentheses indicates Accession Number in GenBank. (TIF) [file pone.0104532.s002.tif]

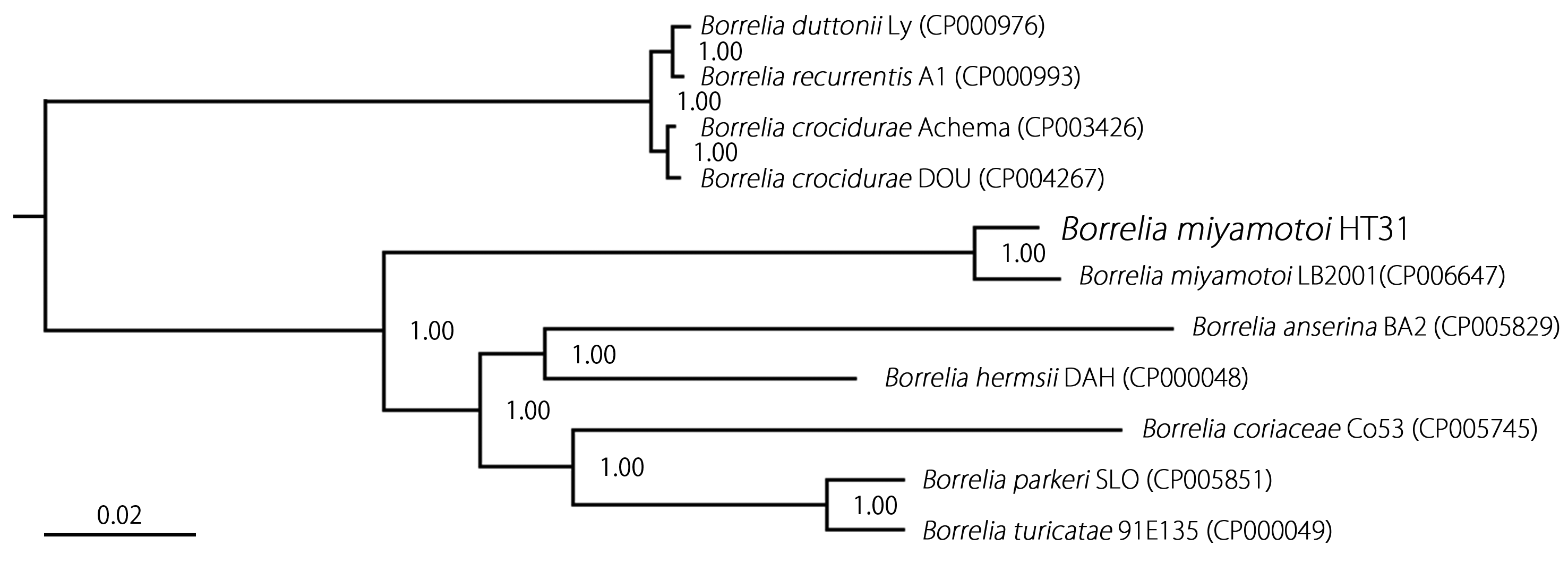

Supplement: Figure S3 — Bayesian phylogenetic inference of concatenated housekeeping gene sequences of RF borreliae. The phylogenetic tree was constructed based on Bayesian phlylogenetic inference as previously described by Margos et al [23]. The posterior probability values of the clades are provided. Bars labeled 0.02 depict 2% divergence. The LD borreliae (ST1 [B. burgdorferi B31], ST84 [B. garinii PBi], ST70 [B. afzelii VS461] were downloaded from the MLST website; www.mlst.net) were used as outgroups (data not indicated). The number in parentheses indicates Accession Number in GenBank. (TIF) [file pone.0104532.s003.tif]
