# Supplementary material for: Tick Surveillance for Relapsing Fever Spirochete Borrelia miyamotoi in Hokkaido, Japan
Source: PLoS One. 2014 Aug 11;9(8):e104532. doi: 10.1371/journal.pone.0104532 (PMC4128717; doi:10.1371/journal.pone.0104532)
Supplement: Table S1 — The composition of BSK-M medium. (DOC) [file pone.0104532.s004.doc]

Table S1. The composition of BSK-M medium.

| Components | Quantity |
| --- | --- |
| HEPES | 3 g |
| Sodium Dehydrogen Citrate | 0.35 g |
| D-(+)-Glucose | 2.5 g |
| Sodium Pyruvate | 0.4 g |
| N-Acetyl Glucosamine | 0.2 g |
| Sodium Bicarbonate | 1.1 g |
| MEM alpha | 5.2 g |
| TC Yeastlate | 1 g |
| Bovine serum albumin, fraction V | 25 g |
| Neopeptone | 2.5 g |
| Young Rabbit serum^*1^ | 40 ml |
| 100 mg/ml Rifampicin in DMSO (If necessary) | 0.34 ml |
| 1N NaOH | Adjust pH7.2 |
| dH_2_0^*2^ | Adjust to 584 ml |

*1 Rabbit serum was incubated at 56ºC 30 minutes before use.

*2 After adjustment to 584ml, medium was filtrated by 0.22 μm PES filter.
