# Supplementary material for: Tick Surveillance for Relapsing Fever Spirochete Borrelia miyamotoi in Hokkaido, Japan
Source: PLoS One. 2014 Aug 11;9(8):e104532. doi: 10.1371/journal.pone.0104532 (PMC4128717; doi:10.1371/journal.pone.0104532)
Supplement: Table S2 — The Primer list used in this study. (DOC) [file pone.0104532.s005.doc]

Table S2. The Primer list used in this study.

| Purpose | Target gene | Primer Name | Sequences (5’ – 3’) | Reference |
| --- | --- | --- | --- | --- |
| MLST | *clpA* | RF_clpA_F | TAAATTTAARCTTGAGGG *1 | In this study *2 |
|  |  | RF_clpA_R | TTAAYAAAAYTATTCAYCTCTTC* | In this study |
|  |  |  |  |  |
|  | *clpX* | RF_clpX_243F | ATATTATTGGRCARGAAGATGC *1 | In this study |
|  |  | clpX_1277R_RF | AGAATTGATTTCATAAAGTTCTTTTG | In this study |
|  |  |  |  |  |
|  | *nifS* | RF_nifS_F | AGGAAAGAYTTYCCTATTYTAAATAAAAC *1 | In this study |
|  |  | RF_nifS_R | ACCCCAATDCCTGTTGGAGCAAGC *1 | In this study |
|  |  |  |  |  |
|  | *pepX* | pepX_222F_RF | ATGAACCATTYYTAATAGC *1 | In this study |
|  |  | RF_pepX_1136R | TTTGARATTGGRCCAATTG *1 | In this study |
|  |  |  |  |  |
|  | *pyrG* | RF_pyrG_F | TTGGTGGTACTGTRGGRGATATGG *1 | In this study |
|  |  | RF_pyrG_R | AAGTTGCATKCCAAGACAAATRCCAAG *1 | In this study |
|  |  |  |  |  |
|  | *recG* | recG_679F_RF | TTTTTTTRCTTCARTTTTTTTCAAG *1 | In this study |
|  |  | RF_recG_1848R | TCTGCTATTTTAAAWCCATC *1 | In this study |
|  |  |  |  |  |
|  | *rplB* | RF_rplB_40F | TCTTTRCGTTATAAGACAAC *1 | In this study |
|  |  | rplB_801R_RF | TTTCGRGTCTTATAYCCCTTAG *1 | In this study |
|  |  |  |  |  |
|  | *uvrA* | uvrA_1216F_RF | TTTATGARRGTTTRATGTCTCG *1 | In this study |
|  |  | uvrA_2235R_RF | TAACATTAAAAGAAAAYCTACC *1 | In this study |
|  |  |  |  |  |
| Real time PCR (primer) | 16S rRNA gene | 16S_RT_F | GCTGTAAACGATGCACACTTGGT | *3 |
|  |  | 16S_RT_R | GGCGGCACACTTAACACGTTAG | *3 |
| Real time PCR (probe) |  | BB_FAM | FAM-TTCGGTACTAACTTTTAGTTAA-MGB | *3 |
|  |  | BM_VIC | VIC-CGGTACTAACCTTTCGATTA-MGB | *3 |
|  |  |  |  |  |
| Conventional PCR/ Sequencing | 16S rRNA gene | rrs-F1(3-26) | ATAACGAAGAGTTTGATCCTGGCT | *4 |
|  |  | rrs-F2(682-703) | GGTGTAAGGGTGGAATCTGTTG | *4 |
|  |  | rrs-R3(749-768) | TTTCGTGACTCAGCGTCAGT | *4 |
|  |  | rrs-R4(1542-1520) | AAAGGAGGTGATCCAGCCRCACT *1 | *4 |
|  |  |  |  |  |
|  | *glpQ* | glpQ-F | GGTATGCTTATTGGTCTTC | *5 |
|  |  | glpQ-R | TTGTATCCTCTTGTAATTG | *5 |
|  |  |  |  |  |
|  | *flaB* | BflaPAD | GATCARGCWCAAYATAACCAWATGCA *1 | *4 |
|  |  | BflaPDU | AGATTCAAGTCTGTTTTGGAAAGC | *4 |

*1: R is A or G, Y is C or T, D is G, A or C, K is G or T, and W is A or T.

*2: The PCR cycle was denatured at 94°C for 30s, annealed at 50°C for 30s and extended at 72°C for 30s for 35 cycles.

*3: Barbour AG, Bunikis J, Travinsky B, Hoen AG, Diuk-Wasser MA, Fish D, Tsao JI (2009) Niche partitioning of *Borrelia burgdorferi* and *Borrelia miyamotoi* in the same tick vector and mammalian reservoir species. Am J Trop Med Hyg 81: 1120-1131.

*4: Takano A, Goka K, Une Y, Shimada Y, Fujita H, Shiino T, Watanabe H, Kawabata H (2010) Isolation and characterization of a novel *Borrelia* group of tick-borne borreliae from imported reptiles and their associated ticks. Environ Microbiol 12: 134-146.

*5: Bacon RM, Pilgard MA, Johnson BJ, Raffel SJ, Schwan TG (2004) Glycerophosphodiester phosphodiesterase gene (*glpQ*) of *Borrelia lonestari* identified as a target for differentiating *Borrelia* species associated with hard ticks (Acari:Ixodidae). J Clin Microbiol 42: 2326-2328.
