# Supplementary material for: Tick Surveillance for Relapsing Fever Spirochete Borrelia miyamotoi in Hokkaido, Japan
Source: PLoS One. 2014 Aug 11;9(8):e104532. doi: 10.1371/journal.pone.0104532 (PMC4128717; doi:10.1371/journal.pone.0104532)
Supplement: File S1 — The sensitivity and specificity of the qPCR. (DOC) [file pone.0104532.s006.doc]

**Supporting Information**

**The sensitivity and specificity of the qPCR**

To determine the sensitivity of the qPCR, an external standard template containing 10^1^ to 10^8^ copies of the *16S rDNA* fragment of *B. miyamotoi* HT31 [1] and *Borrelia garinii* HkIP2 [2] were included in a run. In order to establish the external standard plasmids, a fragment of *16S rDNA* (70bp or 69bp, respectively) was amplified by PCR using the same set of primers for qPCR, and cloned as previously described [3]. To validate the sensitivity among three machines, the same external standard template was used. For analysis of qPCR, the threshold line was set at 0.4 (ABI PRISM 7000 system and LightCycler 480 systemII) or 0.2 (ABI StepOne system). To evaluate specificity of the qPCR, a total of 37 borrelial strains were used: 7 reptile associated borreliae (2 of *Borrelia* sp. tAG, *Borrelia turcica* IST7^T^, *Borrelia* sp. Tortoise14H1, *Borrelia* sp. BF-16, *Borrelia* sp. TA2 and *Borrelia* sp. Tick98M) [3, 4], 13 LD borreliae (*Borrelia burgdorferi* B31^T^, *B. garinii* 20047^T^, *Borrelia afzelii* VS461^T^, *Borrelia valaisiana* VS116^T^, *B. valaisiana* Am501, *Borrelia tanukii* Hk501^T^, *Borrelia turdi* Ya501^T^, *Borrelia sinica* CMN3^T^, *Borrelia bissettii* DN127^T^, *Borrelia japonica* HO14^T^, *Borrelia andersonii* 21123, *Borrelia spielmanii* PsigII 27/6 and *Borrelia lusitaniae* PotiB2^T^) and 5 RF borreliae (*B. miyamotoi* HT31^T^, *B. miyamotoi* FR64b, *Borrelia hermsii*, *Borrelia duttonii* Ly and *Borrelia coriaceae* Co53^T^). Besides these strains, hard-tick borne RF borreliae, *Borrelia* sp. AGRF, were also used [5].

**References**

1. Fukunaga M, Takahashi Y, Tsuruta Y, Matsushita O, Ralph D, McClelland M, Nakao M (1995) Genetic and phenotypic analysis of *Borrelia miyamotoi* sp. nov., isolated from the ixodid tick *Ixodes persulcatus*, the vector for Lyme disease in Japan. Int J Syst Bacteriol 45: 804-810.

2. Takano A, Nakao M, Masuzawa T, Takada N, Yano Y, Ishiguro F, Fujita H, Ito T, Ma X, Oikawa Y, Kawamori F, Kumagai K, Mikami T, Hanaoka N, Ando S, Honda N, Taylor K, Tsubota T, Konnai S, Watanabe H, Ohnishi M, Kawabata H (2011) Multilocus sequence typing implicates rodents as the main reservoir host of human-pathogenic *Borrelia garinii* in Japan. J Clin Microbiol 49: 2035-2039.

3. Takano A, Fujita H, Kadosaka T, Konnai S, Tajima T, Watanabe H, Ohnishi M, Kawabata H (2011) Characterization of reptile-associated *Borrelia* sp. in the vector tick, *Amblyomma geoemydae*, and its association with Lyme disease and Relapsing fever *Borrelia* spp. Environ Microbiol Rep 3: 632-637.

4. Takano A, Goka K, Une Y, Shimada Y, Fujita H, Shiino T, Watanabe H, Kawabata H (2010) Isolation and characterization of a novel *Borrelia* group of tick-borne borreliae from imported reptiles and their associated ticks. Environ Microbiol 12: 134-146.

5. Takano A, Sugimori C, Fujita H, Kadosaka T, Taylor KR, Tsubota T, Konnai S, Tajima T, Sato K, Watanabe H, Ohnishi M, Kawabata H (2012) A novel relapsing fever *Borrelia* sp. infects the salivary glands of the molted hard tick, *Amblyomma geoemydae*. Ticks Tick Borne Dis 3: 259-261.
